# Role of Sanger Sequencing in the Early Diagnosis of Infective Endophthalmitis: Experience From a Pilot Study

**DOI:** 10.7759/cureus.96584

**Published:** 2025-11-11

**Authors:** Priti Singh, Samendra Karkhur, Kanika Singh, Rama Tulasi Siri Duddumpudi, Ashvini K Yadav, Debasis Biswas

**Affiliations:** 1 Ophthalmology, All India Institute of Medical Sciences, Bhopal, Bhopal, IND; 2 Regional Virology Laboratory, All India Institute of Medical Sciences, Bhopal, Bhopal, IND; 3 Microbiology, All India Institute of Medical Sciences, Bhopal, Bhopal, IND

**Keywords:** culture-negative infections, endophthalmitis, molecular diagnostics, ocular microbiology, pcr, sanger sequencing, vitreous biopsy

## Abstract

Background

This study aimed to evaluate the role of Sanger sequencing in the early diagnosis of infectious endophthalmitis and compare its diagnostic yield with conventional microbiological methods.

Methodology

A prospective pilot study was conducted at a tertiary eye care center over a period of one year, including 11 patients with clinically suspected endophthalmitis. Vitreous samples were subjected to Gram staining, KOH wet mount, and aerobic/fungal culture. Parallel molecular testing was performed using 16S rRNA PCR and Sanger sequencing. Diagnostic yield, turnaround time, and clinical relevance were compared.

Results

Gram stain and KOH wet mount were positive in 1/11 (9.1%) cases each, while cultures were sterile in 9/11 (81.8%) and showed contaminants in 2/11 (18.2%). PCR amplification succeeded in 6/11 (54.5%) samples, with Sanger sequencing identifying *Achromobacter* (one case) and *Bacillus* spp. (three cases). Overall, molecular diagnostics established a likely bacterial etiology in 6/11 (54.5%) patients. Sequencing provided results within 24-48 hours, versus 5-7 days for culture.

Conclusions

Sanger sequencing significantly improved diagnostic yield and reduced turnaround time compared with conventional techniques. Although not a replacement for culture, it represents a valuable adjunct in managing culture-negative endophthalmitis. Larger multicentric studies are warranted to validate its routine clinical role.

## Introduction

Endophthalmitis is one of the most feared ocular emergencies, often leading to profound visual impairment or even loss of the eye if not diagnosed and treated promptly. It can arise exogenously following intraocular surgery, penetrating trauma, or intravitreal injections or endogenously through hematogenous spread of systemic infection. Despite advances in prophylaxis, the incidence remains clinically significant worldwide, with devastating consequences in resource-limited settings where diagnostic facilities are scarce [1,2].

Precise and prompt identification of the causative organism is essential to optimize therapy. However, conventional methods such as Gram staining, potassium hydroxide (KOH) wet mount, and microbial culture frequently yield poor results. Culture positivity in endophthalmitis varies widely from 30% to 70% in most large series, and often drops dramatically in patients who have received prior antibiotics or in those with chronic or low-grade infections [3,4]. Moreover, culture requires five to seven days for results, typically delaying empirical decision-making [5].

Recently, molecular diagnostic tests have emerged as a promising alternative to conventional microbiology. Broad-range 16S rRNA PCR, quantitative PCR, and metagenomic sequencing have demonstrated superior sensitivity, even in partially treated cases or when fastidious organisms are involved [6,7]. Studies using high-throughput next-generation sequencing (NGS) report 80-90% positivity rates, compared to less than 50% by culture [3,8,9]. Similarly, nanopore sequencing platforms provide rapid and accurate pathogen identification, with the added advantage of detecting polymicrobial infections and antimicrobial resistance genes [1,8,10].

Sanger sequencing, a long-established molecular tool, has lately been applied in ophthalmic microbiology. Although less high-throughput than NGS, it offers several advantages: it is relatively inexpensive, widely available, and capable of providing actionable results within 24-48 hours. Several studies have shown that Sanger sequencing targeting the 16S rRNA gene can identify bacterial genera in cases where conventional microbiology is negative, thereby improving diagnostic yield [11,12]. It is particularly valuable in situations where sample volumes are small, as in vitreous taps or biopsies, and where culture is often compromised [5,11].

The incorporation of molecular techniques into standard ophthalmic practice is supported by recent research and data. Compared to 28% by culture, Zhu et al. showed that metagenomic sequencing identified pathogens in almost 89% of endophthalmitis patients [3]. Nanopore-based deep sequencing identified organisms in 40% of culture-negative samples and found identical organisms in culture-positive cases, according to Low et al. [8]. Mishra et al. highlighted the use of sequencing as a supplementary tool by concentrating on targeted sequencing for the detection of bacterial DNA in culture-negative endophthalmitis [11]. More contemporary innovations such as CRISPR-Cas-9-based assays would designate the future of swift, on-site molecular testing in ophthalmology [13].

Despite these advances, the role of Sanger sequencing specifically in the real-world diagnostic workflow of endophthalmitis remains underexplored. Most tertiary centers still rely heavily on smear and culture, even though their sensitivity is limited. The necessity of early, trustworthy, and easily accessible diagnostic modalities has been highlighted by studies conducted in various geographical areas [2,5,14,15]. Importantly, molecular methods such as Sanger sequencing may reduce turnaround time and increase yield, giving doctors more confidence to modify empirical therapy.

This gap in evidence provides the rationale for the present pilot study. Prospectively analyzing the vitreous samples from patients with clinically suspected infective endophthalmitis, and comparing the diagnostic yield of conventional Gram, KOH staining, and culture with that of PCR and Sanger sequencing, we sought to determine the feasibility, diagnostic accuracy, and clinical utility of Sanger sequencing in routine ophthalmic practice. Our study represents an early step toward defining the role of Sanger sequencing in the evolving diagnostic landscape of ocular infections.

## Materials and methods

Study design and ethical approval

This was a prospective, observational, pilot study conducted at the Department of Ophthalmology, All India Institute of Medical Sciences (AIIMS), Bhopal, a tertiary ophthalmic care center in central India. The study was reviewed and approved by the Institutional Human Ethics Committee and Institutional Review Board (approval number: IL082). All procedures adhered to the principles outlined in the Declaration of Helsinki. Written informed consent was obtained from all participants or their legal guardians before enrolment. Additional consent was obtained for the use of ocular fluid samples for molecular analysis (PCR and Sanger sequencing). Participation incurred no extra cost to patients, and the standard of care was not compromised.

Study population

Patients presenting with clinically suspected or diagnosed infectious endophthalmitis during the study period of one year were consecutively recruited.

Inclusion criteria

Patients of any age presenting with a clinical diagnosis of infectious endophthalmitis were eligible for inclusion, encompassing both treatment-naïve individuals and those who were previously exposed to antimicrobial therapy. The study included cases of endogenous and exogenous endophthalmitis, such as those arising post-surgically or following ocular trauma. Participation required written informed consent from patients or their legal guardians (in the case of minors), specifically consenting to study enrolment and vitreous sampling via pars plana vitrectomy (PPV) or biopsy.

Exclusion criteria

Cases that had progressed to panophthalmitis, where ocular salvage was not feasible, were excluded from the study, along with patients or guardians who declined consent for PPV or vitreous sampling. Additionally, eyes exhibiting advanced corneal melt or perforation were excluded due to the inability to perform intraocular procedures safely. This exclusion was justified by the poor visual prognosis associated with panophthalmitis and severe corneal compromise, both of which hinder safe vitreous sampling and thereby limit the scope for effective diagnostic and therapeutic interventions.

Study procedure

A total of 11 patients (10 males and 1 female) meeting the inclusion criteria were enrolled in the study. Each patient underwent a comprehensive ophthalmic and systemic evaluation. Ophthalmic assessment included best-corrected visual acuity (BCVA), intraocular pressure measurement, and examination of the anterior and posterior segments using slit-lamp biomicroscopy and indirect ophthalmoscopy, along with extraocular motility testing. Imaging modalities such as B-scan ultrasonography were employed in cases with media opacity, while orbital X-ray or CT was performed when an intraocular foreign body was suspected.

Systemic evaluation comprised routine laboratory investigations, including complete blood count (CBC), random blood sugar, hemoglobin A1c, and viral serology (human immunodeficiency virus (HIV), hepatitis B surface antigen, hepatitis C virus), as well as chest X-ray and screening for systemic risk factors such as diabetes, septicemia, immunosuppression, or malignancy. Pre-anaesthetic evaluation was conducted for patients requiring surgical intervention.

Vitreous samples were collected under aseptic conditions by a trained vitreoretinal surgeon, using either vitreous tap, biopsy, or PPV, depending on clinical indication, with the Alcon Constellation Vision System (Fort Worth, TX, USA). Approximately 200 μL of undiluted vitreous was aspirated via a syringe attached to the vitrectomy cutter before the infusion of balanced salt solution. Samples were immediately transported under cold chain to the microbiology laboratory, where one portion was processed for conventional microbiological evaluation and the remainder preserved for molecular analysis.

Microbiological testing included Gram staining for bacteria, KOH wet mount for fungi, and aerobic and fungal cultures using automated systems, with cultures incubated for up to seven days before being declared sterile. Molecular analysis involved DNA extraction using the QIAamp Viral RNA Mini Kit (Qiagen, Germany), followed by PCR amplification of the broad-range bacterial 16S rRNA gene using the MicroSEQ Full Gene PCR Kit (Thermo Fisher). Amplification was confirmed via electrophoresis on a 2% agarose gel stained with SYBR Safe dye. Purified amplicons were sequenced using the BigDye Terminator v3.1 Cycle Sequencing Kit on a 3500XL Genetic Analyser (Applied Biosystems, Thermo Fisher), and resulting FASTA files were analyzed using NCBI BLAST for homology search and MEGA X software for phylogenetic analysis, with genus-level identification considered clinically significant. In selected cases with strong clinical suspicion of viral etiology, such as HIV-positive patients with known cytomegalovirus (CMV) retinitis, pathogen-specific PCR primers for CMV and herpes simplex virus were additionally employed.

Molecular methods

Each vitreous sample was divided into separate aliquots, and one portion was used for molecular testing under strictly sterile conditions. DNA was extracted using the QIAamp Viral RNA Mini Kit (Qiagen, Germany), following the manufacturer’s protocol optimized for small-volume ocular specimens.

For bacterial detection, the 16S rRNA gene was amplified with universal primers 27F (5′-AGAGTTTGATCMTGGCTCAG-3′) and 1492R (5′-TACGGYTACCTTGTTACGACTT-3′), targeting a ~1,465-bp region of the bacterial genome. Each 25-µL PCR reaction contained 12.5 µL of PCR master mix (Thermo Fisher Scientific), 1 µL of each primer (10 µM), 5 µL of extracted DNA, and 5.5 µL of nuclease-free water. The thermal cycling conditions were initial denaturation at 95°C for five minutes, followed by 35 cycles of denaturation at 95°C for 30 seconds, annealing at 55°C for 30 seconds, and extension at 72°C for one minute, with a final extension at 72°C for seven minutes. Successful amplification was confirmed by running the products on a 2% agarose gel stained with SYBR Safe dye.

Purified amplicons, obtained using the ExoSAP-IT PCR cleanup kit (Applied Biosystems), were then sequenced in both directions using the BigDye Terminator v3.1 Cycle Sequencing Kit on an Applied Biosystems 3500XL Genetic Analyzer. Sequence chromatograms were manually checked for quality, and forward and reverse reads were aligned to generate consensus sequences using MEGA X software. The resulting sequences were compared with reference databases using NCBI BLAST, with ≥97% identity considered indicative of a genus-level match. To ensure reliability, negative extraction and PCR controls were included in each batch to rule out contamination.

Data collection and analysis

Demographic, clinical, and laboratory data were recorded on predesigned proformas. The diagnostic yield was compared among Gram staining, KOH wet mount, culture, and Sanger sequencing. Turnaround times were documented. Descriptive statistics were used to summarize the findings. No formal sample size calculation was undertaken as this was a pilot study.

## Results

Patient demographics

A total of 11 patients with clinically suspected infectious endophthalmitis were enrolled. The mean age was 38.5 ± 23.2 years (range = 5-70 years). There were 10 (90.9%) males and one (9.1%) female. Clinical and demographic details are summarized in Table 1.

**Table 1:** Demographic and clinical profile of patients with infective endophthalmitis. The table summarizes the clinical and demographic characteristics of patients with suspected infective endophthalmitis. VA = visual acuity; HM = hand movements; PL = perception of light; PR = projection of rays; CF = counting fingers; PPV = pars plana vitrectomy; PPL = pars plana lensectomy; SOI = silicone oil injection; PF = phaco-fragmentation; CL = contact lens; HTN = hypertension; DM = diabetes mellitus; CMV = cytomegalovirus; HIV = human immunodeficiency virus

Case	Age (years)	Sex	Predisposing factor	Systemic association	Duration of symptoms	Initial VA	Procedure performed	Treatment given	Final VA
Case 1	60	M	Cataract surgery (2 years prior)	-	2 years	HM+	Vitreous biopsy	Vit + intravitreal antibiotics	2/20
Case 2	70	M	Cataract surgery (1 month prior)	HTN	12 days	HM+	Vitreous biopsy	Vit + intravitreal antibiotics	HM+
Case 3	5	M	Trauma – stone injury	-	10 days	Not recorded	Vitreous biopsy	Vit + intravitreal antibiotics + CL	Not recorded
Case 4	65	M	Endogenous (prostatomegaly, LUTS)	-	2 years	PL+ PR-	Vitreous biopsy	Vit + intravitreal antibiotics	2/20
Case 5	8	M	Trauma – scissor injury	-	4 days	HM+	Primary repair	Vit + intravitreal antibiotics	CF at 3 ft
Case 6	20	M	None identified	-	1 month	HM+	PPV + PPL + SOI	Vitrectomy-based management	HM+
Case 7	47	M	Trauma – iron wire injury	-	15 days	HM+	PPV + PF + SOI	Vit + intravitreal antibiotics	HM+
Case 8	57	M	Endogenous (HIV, CMV infection)	HIV with CMV infection	Not documented	Not recorded	Vitreous biopsy	Supportive/antiviral testing	HM+
Case 9	43	M	Endogenous (liver abscess, dental procedure)	DM, HTN, hypothyroidism, liver abscess	2.5 months	CF at 1 ft	Vitreous biopsy	Vit + intravitreal antibiotics	CF at 1 ft
Case 10	28	M	Trauma – iron particle injury	-	Not documented	PL- PR-	Primary repair	Vit + intravitreal antibiotics	PL- PR-
Case 11	21	F	Endogenous (sepsis, renal failure post-LSCS)	Sepsis, renal failure	1 month	PL+ PR+	PPV	Vit + intravitreal antibiotics	5/60

Predisposing factors and systemic associations

The most common predisposing factor was ocular trauma in four (36.4%) patients, followed by endogenous endophthalmitis in four (36.4%) patients and post-cataract surgery in two (18.2%) patients. One (9.1%) patient presented with no clear identifiable risk factor. Systemic comorbidities were present in four (36.4%) patients, including diabetes mellitus, hypertension, liver abscess with jaundice, HIV with CMV infection, and renal failure with sepsis.

Initial clinical presentation

The duration of symptoms ranged from four days to two years. Presenting visual acuity (VA) varied between counting fingers at 1 ft and perception of light (PL+), with one pediatric patient unable to cooperate for VA testing. Four patients were immunocompromised, with systemic associations including HIV infection, diabetes with sepsis, malignancy, and renal failure. The clinical and demographic characteristics of all patients are summarized in Table 1.

Conventional Microbiological Results

Conventional diagnostic methods demonstrated limited yield. Gram staining was positive in only 1 of 11 (9.1%) cases, revealing Gram-positive cocci and bacilli. KOH wet mount detected melanized hyphal fragments in one (9.1%) case. Aerobic cultures were sterile in nine (81.8%) cases and reported contaminants in two (18.2%) cases, while fungal culture was sterile in all 11 (100%) cases. Overall, conventional microbiology provided actionable information in only 2 of 11 (18.2%) patients. Findings are detailed in Table 2.

**Table 2:** Comparison of diagnostic yields by different methods. The table shows the diagnostic results of conventional methods (Gram stain, KOH, culture) compared with molecular methods (PCR amplification and Sanger sequencing). Sanger sequencing improved diagnostic yield in several culture-negative cases.

Case	Gram stain	KOH wet mount	Culture	PCR amplification	Sanger sequencing result
Case 1	Positive (G+ cocci and bacilli)	Negative	Sterile	Positive	*Bacillus* spp.
Case 2	Negative	Negative	Contaminant	Positive	*Bacillus* spp.
Case 3	Negative	Negative	Sterile	Negative	-
Case 4	Negative	Negative	Sterile	Positive	*Bacillus* spp.
Case 5	Negative	Positive (melanized hyphae)	Sterile	Positive	Achromobacter
Case 6	Negative	Negative	Sterile	Positive	-
Case 7	Negative	Negative	Sterile	Negative	-
Case 8	Negative	Negative	Sterile	Negative	-
Case 9	Negative	Negative	Sterile	Positive	-
Case 10	Negative	Negative	Contaminant	Negative	-
Case 11	Negative	Negative	Sterile	Positive	-

Molecular Diagnostics (PCR and Sanger Sequencing)

**Figure 1:**
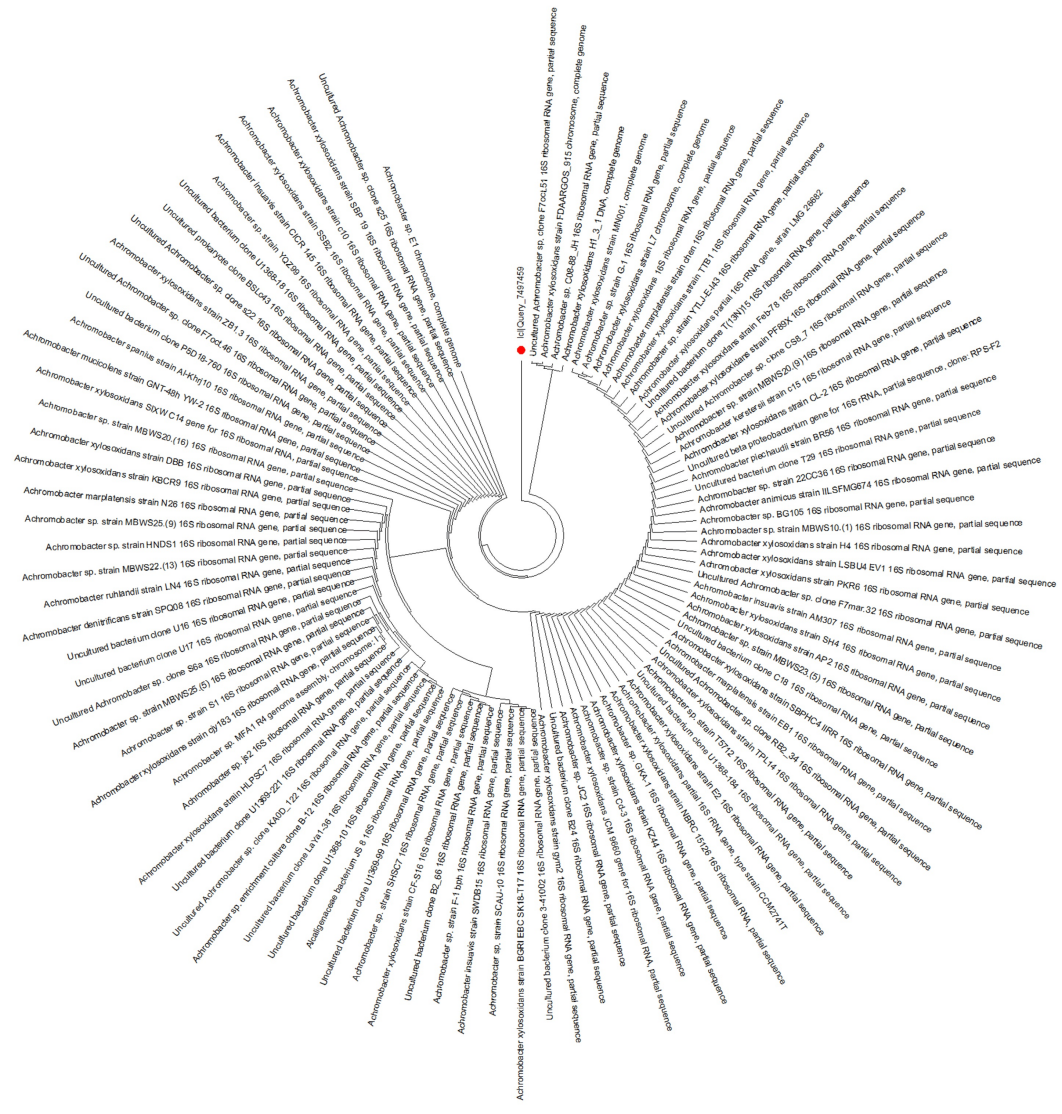
Phylogenetic analysis of 16S rRNA gene sequences obtained through Sanger sequencing where the clinical isolate marked with the red dot showed >90% alignment with sequences belonging to the genus Achromobacter.

**Figure 2:**
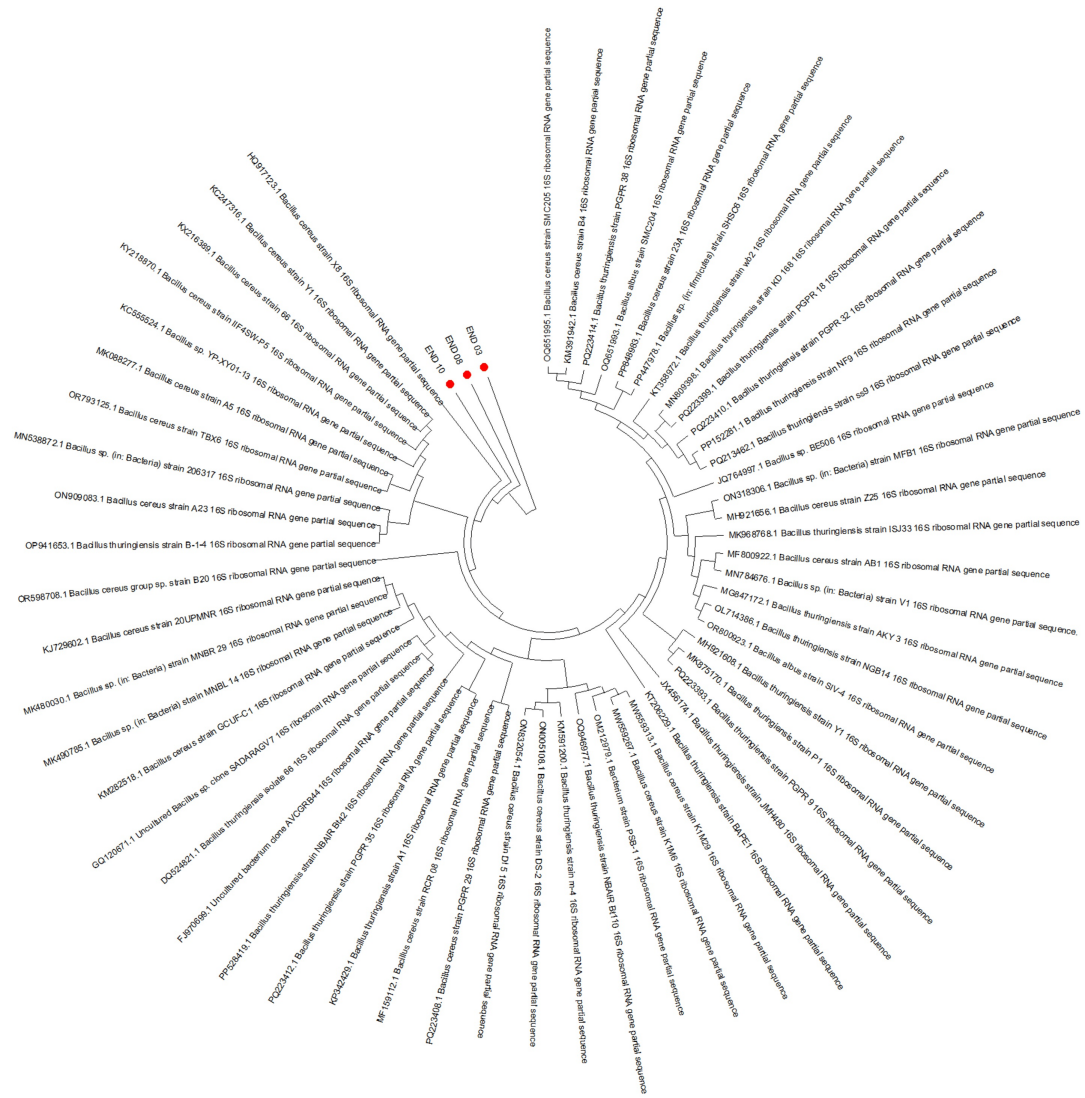
Phylogenetic tree of 16S rRNA gene sequences demonstrating the clustering of three clinical isolates with Bacillus species (marked with red dots).

Of the 11 vitreous samples processed for molecular analysis, PCR amplification of the 16S rRNA gene was successful in six (54.5%) cases. Subsequent Sanger sequencing identified bacterial genera in four of these six (66.7%) cases: *Achromobacter* in one patient and *Bacillus* spp. in three patients (Figures 1, 2). In the HIV-positive patient with known CMV infection, both targeted viral PCR and sequencing were sterile. In total, molecular diagnostics identified a likely bacterial etiology in 6 of 11 (54.5%) patients, with genus-level identification in 4 out of 11 (36.4%) patients. These results are presented in Table 2.

Turnaround Time

Turnaround times varied by modality. Gram stain and KOH yielded results within approximately eight hours, whereas Sanger sequencing provided results within 24-48 hours. Conventional culture required five to seven days for final reporting. A comparison of diagnostic yields across all methods is provided in Table 3.

**Table 3:** Overall diagnostic yield of conventional and molecular methods. The table summarizes the diagnostic yield of each technique. While Gram stain and KOH wet mount identified organisms in only 9.1% of cases each, and cultures remained sterile, molecular methods provided significantly higher yields. PCR amplified bacterial DNA in 54.5% of tested samples, and Sanger sequencing identified bacterial genera in 36.4% of all cases.

Diagnostic Method	Positive cases (n)	Total cases (n)	Yield (%)
Gram stain	1	11	9.1%
KOH wet mount	1	11	9.1%
Aerobic/Fungal culture	0 (sterile/contaminants only)	11	0%
PCR amplification (16S rRNA)	6	11	54.5%
Sanger sequencing (genus-level ID)	4	11	36.4%

Treatment and interventions

Intravitreal antibiotics were administered to nine (81.8%) patients, most commonly a combination of vancomycin (1 mg/0.1 mL) and ceftazidime (2.25 mg/0.1 mL), with clindamycin added in one case. Surgical interventions included primary repair of globe rupture in two (18.2%) patients, PPV with or without lensectomy, phaco-fragmentation, or silicone oil injection in three (27.3%) patients, and vitreous biopsy in six (54.5%) patients. Four immunocompromised patients also required systemic optimization alongside ocular management.

Visual outcomes

Final BCVA ranged from 6/6 in one patient to no perception of light (NPL) in severely affected eyes. The majority of patients demonstrated stabilization or improvement in vision following combined surgical and intravitreal antibiotic therapy.

In this cohort, conventional microbiological methods detected organisms in only 2 of 11 (18.2%) patients. By contrast, molecular diagnostics identified bacterial DNA in six (54.5%) patients and achieved genus-level identification in four (36.4%) patients. Importantly, sequencing provided faster and more clinically useful results than culture, underscoring its feasibility and potential utility as an adjunct in the diagnostic workflow of infectious endophthalmitis.

## Discussion

The present prospective pilot study evaluated the role of Sanger sequencing in the early diagnosis of infective endophthalmitis. Among 11 patients, conventional microbiology provided actionable results in only 18.2% of cases, whereas Sanger sequencing detected bacterial DNA in 54.5% and identified organisms at the genus level in 36.4%. Importantly, sequencing results were available within 24-48 hours compared with 5-7 days for culture. These findings suggest that Sanger sequencing offers a significant advantage in improving diagnostic yield and reducing turnaround time, thereby supporting early therapeutic decision-making in endophthalmitis.

Comparison with conventional microbiology

Culture remains the gold standard for confirming endophthalmitis, yet its limitations are well recognized. Culture positivity in endophthalmitis has been reported to range from 30% to 70%, depending on the clinical setting and prior treatment [4,5]. In the present study, culture was sterile in 81.8% and reported contaminants in 18.2% of cases, consistent with recent reports where culture yields are often depressed by prior antibiotic exposure or low microbial load. Gram stain and KOH wet mount positivity were each limited to 9.1%, reinforcing the limited sensitivity of these rapid techniques. These findings echo prior reports where culture negativity has reached 60-70% in suspected cases, especially in chronic or partially treated infections [4,5].

Molecular diagnostics: global evidence

Recent years have seen molecular diagnostics emerge as powerful tools for pathogen detection in endophthalmitis. Zhu et al. demonstrated that metagenomic sequencing detected pathogens in nearly 89% of patients, compared to only 28% by culture [3]. Similarly, nanopore sequencing studies have shown superior diagnostic yield, identifying pathogens in 100% of culture-positive cases and 40% of culture-negative cases, with genus-level agreement up to 78% [8,10]. Our results, with 54.5% PCR positivity and 36.4% genus-level identification by Sanger sequencing, align with these reports but reflect the inherent limitations of single-target sequencing compared to high-throughput NGS.

Cheng et al. highlighted the application of high-throughput sequencing technologies for endophthalmitis, noting their superior sensitivity and ability to detect polymicrobial infections and antimicrobial resistance genes [6]. Low et al. demonstrated the power of deep metagenomic sequencing using nanopore platforms to identify both common and rare organisms [8]. While Sanger sequencing cannot match the breadth of metagenomics, its lower cost, wider availability, and faster turnaround make it more feasible for resource-constrained centers, especially in developing countries.

Advantages of Sanger sequencing

Several studies confirm that Sanger sequencing provides clinically useful information in culture-negative cases. Mishra et al. demonstrated that broad-range 16S rRNA sequencing improved diagnostic yield significantly compared with culture in suspected cases [11]. Qi et al. further showed that 16S sequencing can delineate bacterial composition in different forms of exogenous endophthalmitis, adding valuable insights for empirical therapy [12]. In our study, sequencing identified *Achromobacter* and *Bacillus* spp., organisms that are often difficult to culture and sometimes dismissed as contaminants. Their detection supports the clinical utility of sequencing in informing empirical treatment.

Another important advantage is turnaround time. While Gram/KOH smears provided results in eight hours, their diagnostic yield was negligible. Sanger sequencing provided results in 24-48 hours, significantly earlier than culture. This is critical in the clinical setting where timely adjustment of empirical therapy may influence visual outcomes. These findings align with recent innovations such as microfluidic real-time PCR systems, which provide rapid multiplex pathogen detection in ocular fluids [9].

Alignment with recent literature

Our results corroborate recent literature emphasizing the role of molecular diagnostics in ocular infections. Jing et al. recently reported the clinical performance of nanopore targeted sequencing in endophthalmitis, demonstrating rapid and accurate detection of pathogenic bacteria [1]. Wang et al. reviewed advances in diagnosis and management, highlighting the growing role of molecular assays in supplementing conventional microbiology [2]. Likewise, Teh et al. emphasized the long-term value of integrating molecular methods into the diagnostic workflow to improve pathogen identification rates [14].

Furthermore, innovative approaches such as CRISPR-Cas assays are being explored for rapid fungal endophthalmitis detection, further underscoring the shift toward molecular solutions [13]. Although Sanger sequencing lacks the multiplexing or polymicrobial detection capabilities of NGS or CRISPR assays, its feasibility in clinical laboratories remains an attractive advantage for pilot implementation.

Clinical relevance and therapeutic implications

The ability of Sanger sequencing to identify bacterial DNA even in culture-negative samples provides clinicians with valuable confidence in continuing or adjusting empirical therapy. In our series, organisms identified at the genus level were sufficient to guide broad-spectrum intravitreal antibiotics. Although antibiotic susceptibility testing is not possible with sequencing alone, the ability to confirm bacterial versus fungal etiology supports rational antimicrobial use, a crucial factor given rising antimicrobial resistance.

Four patients in our study were immunocompromised, including those with HIV, liver abscess, and renal failure. In such patients, culture yields are especially poor due to prior antimicrobial exposure and atypical organisms. Sequencing in these cases demonstrated its value, even though some samples remained negative. This supports previous observations that molecular methods may be less affected by prior therapy than culture, making them particularly valuable in tertiary referral settings where patients often arrive partially treated [4,5].

Strengths of the study

This study is among the first prospective pilot evaluations of Sanger sequencing in endophthalmitis from a tertiary care center in central India. It highlights feasibility, real-world turnaround times, and comparison with conventional methods. The prospective design ensures systematic sample collection and uniform application of techniques. By directly comparing smear, culture, and sequencing on the same samples, we demonstrate the incremental value of molecular diagnostics in the routine workflow.

Limitations

Although this study provides valuable insights into the diagnostic potential of Sanger sequencing in infectious endophthalmitis, certain limitations must be acknowledged. First, the small sample size (n = 11) limits statistical power and external generalizability, as this was designed as a pilot study. While diagnostic yields are clearly presented, 95% confidence intervals have now been added to Table 3 to reflect statistical uncertainty. Second, the study employed only broad-range bacterial 16S rRNA primers, which may have missed fungal or viral etiologies. This limitation is particularly relevant to Case 5, where fungal elements were seen on the KOH mount, but PCR was negative. Broader primer panels, including fungal and viral targets, will be essential in future analyses. Third, two of six PCR-positive samples could not be successfully sequenced, likely due to low DNA concentration or mixed chromatogram peaks suggestive of polymicrobial infection. These technical challenges have now been acknowledged and discussed in context. Fourth, antibiotic susceptibility testing was not possible through sequencing, which limits its use for therapeutic de-escalation despite rapid organism identification. Similarly, a formal cost analysis was not conducted; while the technique is relatively low-cost compared to NGS, feasibility in low-resource settings warrants dedicated evaluation. Despite these limitations, the findings underscore the clinical feasibility and diagnostic advantage of Sanger sequencing as a rapid adjunct to conventional microbiology in real-world ophthalmic settings.

Future perspectives

The results of this pilot study underscore the growing importance of molecular diagnostics in the management of infectious endophthalmitis. Although conventional methods remain the cornerstone of ocular microbiology, their limitations, particularly low culture positivity and delayed turnaround time, necessitate adjunctive approaches [4,5]. Sanger sequencing has shown promise as a rapid, cost-effective tool that can provide clinically actionable results within 24-48 hours, especially in culture-negative cases [11,12]. By enabling earlier confirmation of bacterial etiology, it supports rational antimicrobial use, avoids unnecessary antifungal coverage, and strengthens clinician confidence in therapeutic decisions [2,14].

Looking ahead, future studies should focus on larger, multicentric cohorts to validate these findings across diverse clinical and geographic settings [14,15]. Expanding molecular panels to include fungal, mycobacterial, and viral primers will improve coverage in immunocompromised patients and regions with high fungal prevalence [2,4,13]. Integrating Sanger sequencing into multiplex workflows alongside real-time PCR and CRISPR-based assays could further enhance diagnostic accuracy and speed [9,13]. In parallel, NGS should be explored in specialized centers for its ability to detect polymicrobial infections and antimicrobial resistance genes, providing deeper insights for precision therapy [3,6,8,10].

While NGS may remain resource-intensive, Sanger sequencing represents a widely available, first-line molecular approach that can be implemented in most ophthalmic microbiology laboratories [11,12]. Establishing standardized protocols, building regional sequence databases, and developing collaborative multicentric registries will be critical to ensuring reproducibility and clinical adoption [14,15]. Ultimately, incorporating molecular tools into standard care pathways has the potential to transform the diagnostic and therapeutic landscape of endophthalmitis, leading to earlier intervention, better visual outcomes, and reduced morbidity in this sight-threatening disease [1,2,7,13].

## Conclusions

Our study demonstrated that Sanger sequencing improved diagnostic yield and reduced turnaround time compared with Gram/KOH staining and culture in patients with suspected infectious endophthalmitis. These results align with recent global literature highlighting the role of molecular diagnostics in ocular infections. Although not a replacement for culture, Sanger sequencing represents a valuable adjunct, particularly in culture-negative cases. Wider adoption of molecular diagnostics, complemented by conventional microbiology, will enhance early diagnosis and optimize management of this vision-threatening condition.
